# Population Dynamics of Whiteflies and Associated Viruses in South America: Research Progress and Perspectives

**DOI:** 10.3390/insects11120847

**Published:** 2020-11-28

**Authors:** Renate Krause-Sakate, Luís Fernando Maranho Watanabe, Eduardo Silva Gorayeb, Felipe Barreto da Silva, Daniel de Lima Alvarez, Vinicius Henrique Bello, Angélica Maria Nogueira, Bruno Rossitto de Marchi, Eduardo Vicentin, Marcos Roberto Ribeiro-Junior, Julio Massaharu Marubayashi, Claudia Andrea Rojas-Bertini, Cristiane Muller, Regiane Cristina Oliveira de Freitas Bueno, Marlene Rosales, Murad Ghanim, Marcelo Agenor Pavan

**Affiliations:** 1 Department of Plant Protection, Universidade Estadual Paulista “Julio de Mesquita Filho” (UNESP), Botucatu 18610-034, Brazil; luiswatanabe92@hotmail.com (L.F.M.W.); eduardogorayeb@gmail.com (E.S.G.); felipe.barretods@gmail.com (F.B.d.S.); daniel-alvarez92@hotmail.com (D.d.L.A.); vhbello@hotmail.com (V.H.B.); axnogueira@hotmail.com (A.M.N.); evicentin@gmail.com (E.V.); marcosrrjr@gmail.com (M.R.R.-J.); juliommagro@hotmail.com (J.M.M.); regiane.bueno@unesp.br (R.C.O.d.F.B.); ma.pavan@unesp.br (M.A.P.); 2 Facultad de Agronomía e Ingeniería, Pontificia Universidad Católica de Chile, Forestal, Vicuña Mackena, 4860, Macul, Santiago 7820436, Chile; carojas12@uc.cl (C.A.R.-B.); irosalesv@uc.cl (M.R.); 3 Gulf Coast Research and Education Center, University of Florida, Wimauma, FL 33598, USA; bruno_dmarchi@hotmail.com; 4 CortevaTM Agrisciences, Mogi Mirim 13814-000, Brazil; cristiane.muller@corteva.com; 5 Department of Entomology, Institute of Plant Protection, The Volcani Center, Rishon LeZion 7505101, Israel; ghanim@volcani.agri.gov.il

**Keywords:** *Bemisia tabaci*, *Trialeurodes vaporariorum*, begomovirus, crinivirus, carlavirus

## Abstract

**Simple Summary:**

Whiteflies are one of the most important and widespread pests in the world. In South America, the currently most important species occurring are *Bemisia afer,* *Trialeurodes vaporariorum,* and the cryptic species Middle East-Asia Minor 1, Mediterranean, and New World, from *Bemisia tabaci* complex. The present review compiles information from several studies conducted in South America regarding these insects, providing data related to the dynamics and distribution of whiteflies, the associated viruses, and the management strategies to keep whiteflies under the economic damage threshold.

**Abstract:**

By having an extensive territory and suitable climate conditions, South America is one of the most important agricultural regions in the world, providing different kinds of vegetable products to different regions of the world. However, such favorable conditions for plant production also allow the development of several pests, increasing production costs. Among them, whiteflies (Hemiptera: Aleyrodidae) stand out for their potential for infesting several crops and for being resistant to insecticides, having high rates of reproduction and dispersal, besides their efficient activity as virus vectors. Currently, the most important species occurring in South America are *Bemisia afer, Trialeurodes vaporariorum,* and the cryptic species Middle East-Asia Minor 1, Mediterranean, and New World, from *Bemisia tabaci* complex. In this review, a series of studies performed in South America were compiled in an attempt to unify the advances that have been developed in whitefly management in this continent. At first, a background of the current whitefly distribution in South American countries as well as factors affecting them are shown, followed by a background of the whitefly transmitted viruses in South America, addressing their location and association with whiteflies in each country. Afterwards, a series of management strategies are proposed to be implemented in South American fields, including cultural practices and biological and chemical control, finalizing with a section containing future perspectives and directions for further research.

## 1. Introduction

The extensive territory and suitable climate are conducive for the South America region production of a wide range of agricultural commodities. The vast unexploited agricultural area suggests this region will keep playing a significant role in global food production in the future. Nevertheless, as the climate is suitable for cultivation, it is also suitable for threats to agriculture. Among them, there is the whitefly insect group (Hemiptera: Aleyrodidae), which includes more than 1500 species in approximately 161 genera [1].

**Figure 1:**
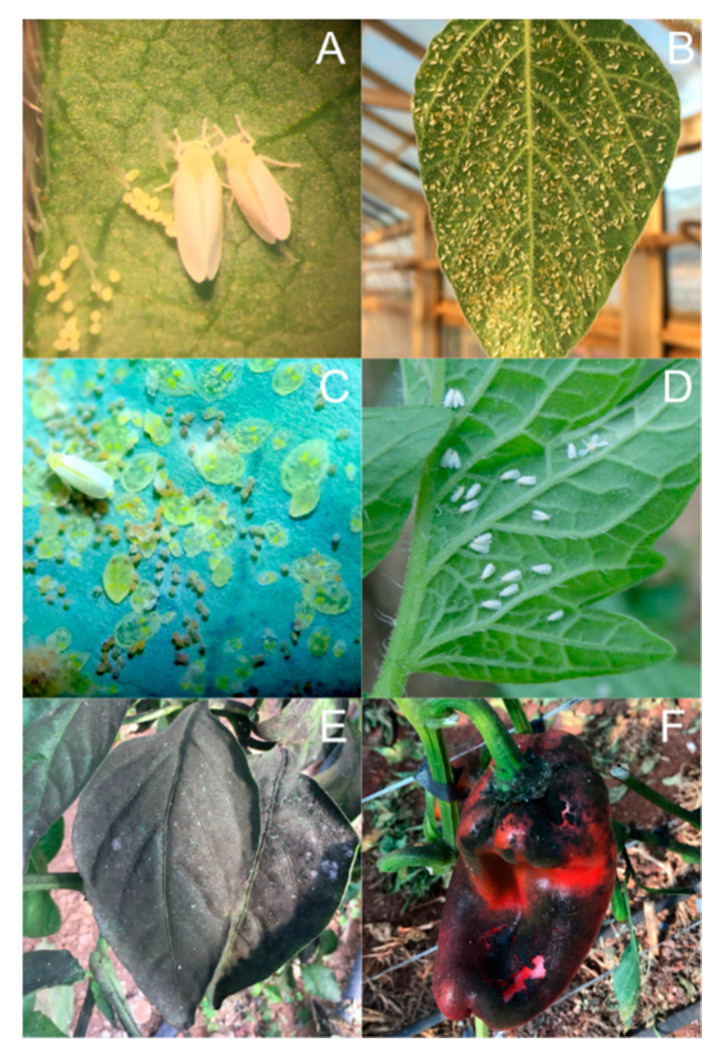
(**A**) Eggs and adults of *Bemisia tabaci*. (**B**) A large infestation of *B. tabaci* on the abaxial side of the soybean leaflet. (**C**) Different life stages of *Bemisia tabaci* colonizing and feeding on a cabbage leaf. (**D**) Adults of *Trialeurodes vaporariorum* feeding on the abaxial side of a tomato leaf. (**E**,**F**) Sooty mold covering sweet pepper leaves and fruits.

Among whitefly species, only a few of them stand out for damaging crops by direct damage and/or virus transmission in South America, including *Bemisia tabaci* (Gennadius) species complex (Figure 1A–C), *Bemisia afer* (Priesner & Hosny) *sensu lato*, and *Trialeurodes vaporariorum* (Westwood) (Figure 1D) [2,3,4,5,6], contributing to losses of over US$ 1 billion per year globally [7,8]. The direct damage occurs by phloem-feeding and the excretion of the honeydew on leaves and fruits, serving as a substrate for the growth of sooty mold that covers the surfaces (Figure 1E,F) and interferes with photosynthesis, reducing hosts productivity as well fruit and fiber quality [8].

However, virus transmission is the main damage caused by whiteflies in agriculture, especially by *B. tabaci* species complex, transmitting viruses from the genera *Begomovirus*, *Carlavirus*, *Crinivirus*, *Ipomovirus*, *Torradovirus,* and *Polerovirus*, and the *T. vaporariorum*, transmitting viruses from the genera *Crinivirus* and *Torradovirus* [5,9,10,11]. These two whitefly species stand out as pests on a worldwide scale. They are small insects, around 1–2 mm (*B. tabaci*) and 1–3 mm *(T. vaporariorum*) (Figure 1C,D), which have three stages in their life cycle, beginning with egg-laying, usually on the abaxial side of the leaves, that lasts approximately from four to seven days, followed by the nymphal stages. The nymphal stages have three phases, beginning with first-instar nymphs that move on the leaf searching for a spot to feed and develop to the second, third, and fourth instars, which are immobile, with atrophied legs and antennae, acquiring red eyes during the fourth instar phase [12]. Finally, from the fourth-instar nymph, the adults emerge with opaque-shape wings covered with a whitish wax, which is related to the common name of “whitefly” [8,12]. The whitefly may harbor endosymbiotic bacteria, which can contribute to their ecology and biology. The endosymbionts colonizing *B. tabaci* are classified as primary (obligatory) and secondary (facultative). *Portiera aleyrodidarum* is a primary endosymbiont, being necessary for survival and reproduction due to the synthesis of essential amino acids that complement the insect diet [13,14,15,16,17]. *Hamiltonella*, *Rickettsia*, *Arsenophonus*, *Wolbachia*, *Cardinium*, *Fritschea,* and *Candidatus* Hemipteriphilus asiaticus (also known as the *Orientia*-like organism–OLO) are secondary, being not essential for insect persistence [8,14,15,18,19,20,21]. However, they may play an important role in the biology, behavior, plant viruses transmission, susceptibility to insecticides, and thermotolerance of their insect hosts [8,15,22,23,24]. Moreover, these organisms can be transmitted horizontally or vertically from one whitefly to another, contributing to creating variation on their frequency and localization inside the insect, according to whitefly life stages (egg, nymph, and adult) and populations [15].

The *B. tabaci* complex has been reported as a super vector of viruses worldwide, transmitting numerous species to solanaceous, fabaceous, cassava, cotton, and several weeds [11,25,26,27]. Additionally, other factors, such as their strong polyphagia, insecticide resistance of some populations, the development of vast populations on plants, and long-distance spreading by infested plant materials or wind currents contribute to their status as one of the world’s most economically important agricultural pests [11,15]. *Bemisia tabaci* has also been listed as a top 100 invader pest worldwide by the IUCN/SSC Invasive Species Specialist Group (http://www.issg.org) and the Global Invasive Species Database (http://www.issg.org/database/welcome/). It was also listed as one of the most important invader pests in Brazil (https://www.gov.br/agricultura/pt-br/assuntos/sustentabilidade/tecnologia-agropecuaria/recursos-geneticos-1/especies-introduzidas) by the Ministry of Agriculture, Livestock, and Supply (MAPA).

In the 1950s, the concept of *B. tabaci* biotypes and host races were proposed after the discovery that morphologically indistinguishable populations exhibited measurably different biological features with respect to host range, host–plant adaptability, and plant virus-transmission capabilities [28,29,30]. Last decade, by using the molecular analysis of the mitochondrial cytochrome oxidase subunit I (mtCOI) gene, *B. tabaci* was classified as a complex of cryptic species [15,31]. According to the information provided by this single-gene genotyping approach, *B. tabaci* is considered a complex of 11 well-defined high-level groups containing at least 44 distinct species [15,31,32]. Among them, the Middle East-Asia Minor 1 (MEAM1, former B biotype), Mediterranean (MED, former Q biotype), and New World (NW, former A biotype) are found in South America [33,34,35]. Despite some authors arguing about whether the definition as biotypes, races, or species is correct [36], in this review, we will consider the generally accepted definition of cryptic species.

Another whitefly that is widely distributed and drives concern for agriculture is the *T. vaporariorum*, also known as the greenhouse whitefly. In South America, it has been reported as a pest, especially under mild conditions or elevated altitude (e.g., Andes region, Uruguay, North of Chile, South of Argentina, and South of Brazil). These species have been important pests of many agricultural and ornamental plants under greenhouse and field conditions in practically all of South America [33,35,37,38,39,40,41].

The South American territory is located, for the most part, between the Tropics of Capricorn and Cancer, in which appropriate conditions for whiteflies’ development occur throughout the year. However, different climate conditions may affect whitefly performance, dispersion, and influence the predomination and spread of viruses, which can change from country to country. Thus, the knowledge of the occurrence and distribution of whitefly species, their endosymbionts, and their associated viruses are of extreme value in directing management practices and programs. Therefore, this review provides updated information about the distribution and dynamics of whiteflies species in South America, showing that *B. tabaci* MEAM1 and MED species and *T. vaporariorum* are a continuous concern on greenhouse and field-grown crops. The emergence and outbreaks of viral diseases are also increasing and are closely related to each whitefly species. Lastly, management strategies based on host plants, ecological niches, insecticides, and alternative control were discussed to keep whiteflies under the economic damage threshold.

## 2. Whitefly Dynamics and Distribution in South America

### 2.1. Historical Perspective of Native and Exotic Whitefly Species Throughout the Years

In South America, whitefly dynamics and distribution have changed during the decades, bringing new concerns for agriculture. Among the economically important species, the greenhouse whitefly (*T. vaporariorum*), *B. afer,* and four cryptic species of *B. tabaci* complex (MEAM1, NW group, and MED) are currently present in South America.

*Trialeurodes vaporariorum* is a polyphagous pest that can damage hosts from more than 80 botanical families [42]. This whitefly is an important pest of greenhouse-cultivated ornamentals and vegetable crops, with some outbreaks also occurring in the field [43]. Its importance increased at the beginning of the 1990s, when it was characterized as a vector of two criniviruses in the United States, tomato infectious chlorosis virus and tomato chlorosis virus, as well as at the beginning of the 2000s, when Salazar and colleagues described this whitefly as the vector of the potato yellow vein virus [12,44].

Westwood firstly described *T. vaporariorum* during the middle of the 19th century. Its correct original region is not well known but is most likely endemic from subtropical American regions, such as southern Brazil or Mexico [42]. It was only officially reported in South America during the 1960s and is present in Argentina, Chile, Colombia, Ecuador, Guiana, Peru, and Brazil [45]. Later, two other reports occurred in the early 2000s in Venezuela and Uruguay [46,47], and finally, in 2016, this whitefly was detected in Bolivia [48].

Within the *Bemisia* genus, *B. afer* is restricted and is particularly a problem in Peruvian lowlands, where it was described for the first time in 2000, following severe outbreaks that occurred in sweet potato [49]. Its importance is associated with transmitting the sweet potato chlorotic stunt crinivirus to this crop, which is very important for the Peruvian coastal regions [3].

*Bemisia tabaci* was officially reported in the Americas in the 1920s [50]. Until the 1970s, this insect was characterized as a secondary pest in South America, infesting mainly weeds, causing sporadic outbreaks in common beans (*Phaseolus vulgaris*), and eventually transmitting viruses, such as *Bean golden mosaic virus* (Begomovirus) [51]. The increase in soybean monocropping area in Brazil combined with pesticide use and no crop-free period contributed to whitefly outbreaks since the mid-1970s [52,53,54] (20, 31, 32). The indigenous whiteflies from the Americas, known as biotype A, would be later classified in the New World group, based on mtCOI analysis [35,37,55]. These whiteflies are currently present in Argentina, Brazil, Bolivia, Colombia, and Venezuela [34].

The scenario dramatically changed at the beginning of the 1990s, with the introduction of the exotic MEAM1 species (biotype B) in South America, associated with different hosts, such as common beans, soybean (*Glycine max*), cotton (*Gossypium* spp.), tomato (*Solanum lycopersicum*), peppers (*Capsicum* spp.) cucurbits, and ornamental plants [52]. In Brazil, for example, the first outbreaks related to MEAM1 occurred in 1991, in São Paulo state, probably associated with the international ornamental plant trading market and migration of insects to crops from surrounding areas [56]. Similarly, in Chile, two introductions occurred, firstly in 1998, associated with infested alfalfa plants from the United States, and later in 1999, when new outbreaks occurred in ornamental plants from central and northern Chilean regions [41].

In Argentina, it was also believed that MEAM1 was introduced associated with the trading of ornamental plants [57], but there is no official evidence to confirm this statement. Extensive surveys were performed between 1994 and 1999, with *B. tabaci* being confirmed as the predominant species. However, its diversity was assessed only in 2003, when a mtCOI analysis revealed the presence of MEAM1, infesting cotton, eggplant (*Solanum melongena*), tobacco (*Nicotiana tabacum*), tomato, and an ornamental from the *Zinnia* genus [57]. In Peru, it is known that *B. tabaci* is present since 1993, but at that time, no molecular analysis was performed [58]. Molecular data reporting the presence of MEAM1 in Peru was only published in 2017, from a sample collected in 2000 in the Cañete Valley [59]. In addition to this report, there is one more MEAM1 nucleotide sequence on Genbank (https://www.ncbi.nlm.nih.gov/genbank) collected in Peru from a direct submission of 2019 (LR535722).

In Colombia, there are also no clues about the introduction route of MEAM1. It was first identified in Sucre, Córdoba, and Atlântico’s northwestern departments along the Atlantic coast in 1997, infesting tomato, cotton, and soybean [60]. These findings led to the creation of a new project dedicated to the study of whiteflies in the Andean tropical highlands, which subsequently identified MEAM1 along with all departments in the Atlantic coast of Colombia, as well as in Cauca valley, Huila, and Cundinamarca, associated with common beans, tomato, potato, and other horticultural crops [61]. The same project also found MEAM1 in the pacific coastal regions of Ecuador, infesting poinsettia (*Euphorbia pulcherrima*), soybean, and several horticultural crops as in tomatoes and cucurbits from Venezuelan regions of Lara and Aragua [61,62].

The last cryptic species introduced in South America was *B. tabaci* MED, which is also a major pest worldwide, causing massive damage to several crops. The first record in the Americas was in the United States [63], and years later, it was reported in Mexico [64] and Guatemala [65]. In South America, MED was first identified in the region of Rio de la Plata, located in the Argentina–Uruguay border, infesting red pepper and cucurbits [38]. Later on, it was reported in Brazil at Barra do Quaraí in 2013, along the border with Uruguay, infecting greenhouse-cultivated bell peppers [66]. Subsequently, a new invasion occurred in 2015 in São Paulo state, coming through the international trade of ornamental plants [67], and quickly spread across the South, Southeast, and Midwest regions of Brazil [35].

### 2.2. Factors Related to Whitefly Dynamics, Distribution, and Pest Status

Insect behavior and development may be affected by different factors, such as temperature, the presence of endosymbionts, host plants, associated viruses, and management. For whiteflies, these factors have a substantial impact on the predominance and establishment.

Temperature is one of the most critical parameters for insect development, affecting their performance, which will influence their colonization, behavior, and distribution [68,69,70]. Studies from Colombia showed that *T. vaporariorum* has better longevity and oviposition under mild conditions (19–22 °C) and worse at 26 °C [40]. In China, it was revealed that adults of MEAM1 endure at higher temperatures (41–43 °C), surviving more than *T. vaporariorum* [71]. The high temperature also reduces the oviposition, hatchability, number of adults generated from eggs, and the percentage of female ratio for both whiteflies, but affects the *T. vaporariorum* more heavily [71]. Moreover, the temperature can also have different effects on gene regulation depending on the whitefly species, expressing and ensuring advantages for the insect. For example, researchers from China showed that the temperatures for induction of the heat shock protein expression were 2 to 6 °C lower for *T. vaporariorum* than those for *B. tabaci* MEAM1 [72], supporting the preference of *T. vaporariorum* and *B. tabaci* MEAM1 for mild and high temperatures, respectively. Other researchers in China revealed that the hsp23 and hsp70 genes might play an essential role in the MEAM1 survival against high temperature, whereas they are higher in females in temperatures from 37.5 °C to 42 °C and in males at 44 °C [73]. These conditions can alter whitefly biology, influencing directly their performance in the field, and may select some species according to their adaptation.

These results are intimately related to whitefly current distribution in South America, where *B. tabaci* species complex is more important in tropical regions, whereas *T. vaporariorum* has a more significant influence in the subtropical and temperate regions, which seems logical, regarding their origin.

In Chile, *B. tabaci* MEAM1 occurrence is limited to the northernmost region of Arica y Parinacota, with warmer temperatures, while *T. vaporariorum* is more critical in central colder regions [74]. Despite also being introduced in the central region, MEAM1 was eradicated, and the climate along with the existence of the Atacama desert acting as a natural barrier seem to be the main reasons for the non-migration of MEAM1 to the southernmost regions [74].

In Brazil, *T. vaporariorum* is also restricted to South and Southeast regions, which have milder temperatures than the rest of the country [35]. *Bemisia tabaci* is also present in those regions, although it is much less harmful than in tropical regions, causing significant damage sporadically.

In Argentina, the *B. tabaci* complex was identified in several northern states until the latitude of La Plata, with reports of the NW group, MEAM1, and MED [33,37,38,75]. Despite this, *T. vaporariorum* is considered the predominant species in this country [76], although there is no availability of detailed surveys with its correct distribution.

Additionally, other countries such as Venezuela, Colombia, Peru, Ecuador, and Bolivia have the influence of the Andes Mountains in the climate, giving some regions temperate or subtropical climates, regardless of their latitude, also influencing whitefly distribution. A survey in Colombia and Ecuador showed the presence of only *T. vaporariorum* in highlands (1000 to 3000 m), and only *B. tabaci* in lowlands (0 to 400 m), with the presence of both only in the mid-altitude valleys (400 to 1000 m), but with a higher proportion of *T. vaporariorum* [61]. These findings were later supported by another survey in Cundinamarca, Colombia, revealing *T. vaporariorum* in most sampled locations. This study included locations varying from 653 to 2680 m in altitude, in which MEAM1 was found only from 653 to 1940 m, while *T. vaporariorum* was found in every municipality tested [77]. Additionally, although present, MEAM1 was always detected coexisting with *T. vaporariorum*, indicating that this whitefly is more distributed and may predominate under these conditions [77].

Moreover, the endosymbiont may also play an important role in the insect’s biology and thermotolerance. For example, in China, the MED population that had *Hamiltonella* laid more eggs, increased nymphal survival, and developed quickly in comparison to an *Hamiltonella*-free MED population when reared on cotton at 26 ± 2 °C [78]. In the USA, a *Rickettsia*-infected population of *B. tabaci* (unknown species) produced more adults, exhibited better survival rate, faster development times, and a higher proportion of females than a *Rickettsia*-free population [79]. Additionally, in Israel, the MEAM1 population with *Rickettsia* showed higher tolerance to heat shock when exposed to 32, 37, and 47 °C than a *Rickettsia*-free population [23]. Thus, the endosymbionts influence may also happen in South America, but lack of information does not allow any confirmation.

The endosymbionts are also evolving according to the whitefly species. A survey among MEAM1 populations collected between 2010–2017 in Brazil showed that they harbor the secondary endosymbionts *Hamiltonella*, *Rickettsia*, *Cardinium*, *Wolbachia,* and *Fritschea*, but with a predominance of *Hamiltonella* and *Rickettsia* [14,35,80]. A MEAM1 population from Uruguay showed co-infection with *Rickettsia*, *Wolbachia,* and *Cardinium* [19]. There is a high variability of secondary endosymbionts in MED populations from Brazil with several combinations of *Hamiltonella*, *Rickettsia*, *Wolbachia*, *Cardinium,* and *Arsenophonus* [35]. For New World populations, all endosymbionts combination except *Rickettsia* has been found [14,35], and in Brazilian populations of *T. vaporariorum*, only *Arsenophonus* has been identified [14,35]. Moreover, a set of endosymbiont may be variable, changing from region to region, as well as by whitefly species, but may steady after years of coevolution [18,19,81,82,83,84,85,86,87,88].

The host can also influence whitefly distribution by affecting their performance. For example, in tomato, MEAM1 has better performance than *T. vaporariorum*, having higher oviposition, hatchability, adult emergence, and survival rate, helping MEAM1 establish first in the field predominate over *T. vaporariorum* [89]. A study revealed that MEAM1 displaced MED on tomato and cabbage, while MED predominated on sweet pepper in China [90]. In Brazil, a similar study was performed, including the main crops grown in this country, showing that when reared on sweet pepper, MED displaced MEAM1, whereas MEAM1 displaced MED on tomato [91]. The recent outbreaks of MED in sweet pepper reported in São Paulo and the Paraná States of Brazil confirmed the previous results [92]. Additionally, when reared on common beans, MED also displaced MEAM1, but when reared on soybean or cotton, both whiteflies coexisted, without one dominating over the other [91]. It is essential to highlight that, in Brazil, soybean, cotton, and common beans are always grown close to each other, and therefore the common beans might serve for increasing the MED population in the field, changing whitefly proportion in the subsequent crops [91].

Moreover, the management, other factors that influence the whitefly dynamics, and distribution will be discussed afterward in this manuscript.

## 3. Whitefly-Transmitted Viruses in South America

Plant viruses are present on all major agronomic important crops, representing losses in yield often above 60% and continuous socio-economic threats to global food security [93]. Insects transmit almost 75% of plant viruses, and those that have evolved their piercing–sucking mouthparts to feed in the phloem are the most common and widespread vectors [4]. Transmission of plant viruses is the most critical damage caused by whiteflies. Most of these viruses are emerging worldwide, of which about 94% belong to the *Begomovirus* genus, 3% to the *Crinivirus* genus, and the remaining 3% are in the *Carlavirus*, *Ipomovirus*, *Polerovirus*, and *Torradovirus* genera [94]. *Ipomovirus* and *Torradovirus*, as far as we know, have not been reported in South America to date, and they will not be addressed in this review.

The genus *Begomovirus* in the family *Geminiviridae* comprises 424 approved species and is the largest genus of whitefly-transmitted viruses. Begomoviruses are transmitted persistently by *B. tabaci*, and most are restricted to the phloem of their hosts. Based on genome organization, phylogenetic relationships, and geographical distribution, species in the genus *Begomovirus* are divided into ”Old World” (OW), occurring in Europe, Africa, Asia, and Australasia, and ”New World” (NW), occurring in the Americas [95,96]. The Americas have the highest incidence and diversity of begomoviruses globally, and the distribution and incidence of begomoviruses in South America are directly associated with the dynamics of their insect vector. The main crops affected by begomoviruses in South America have been common bean, tomato, and sweet and hot peppers.

Species in the genus *Carlavirus* (Family *Betaflexiviridae*) are mostly aphid-transmitted, but cowpea mild mottle virus (CPMMV) and melon yellowing-associated virus (MYaV) are two species currently known to be transmitted by species from the *B. tabaci* cryptic species. CPMMV is transmitted by MEAM1, MED, and NW and is bringing concerns to soybean (*Glycine max*) and common bean (*Phaseolus vulgaris*) farmers, as well as MYaV to melon (*Cucumis melo*) growers, which is transmitted by MEAM1 [97,98,99].

Members of the genus *Crinivirus* (family Closteoviridae) are also responsible for causing diseases that have been gaining prominence in crops in South America, mainly in the solanaceous. Criniviruses are restricted to phloem and transmitted in a semi-persistent way by *B. tabaci* cryptic species MEAM1 (biotype B) and MED (biotype Q), as well as by *T. vaporariorum* and *T. abutilonea*, which are recognized as the primary vectors [5,100]. Studies have shown that criniviruses can be retained in the vector for up to 12 days, depending on the virus and vector species [101,102].

The genus *Polerovirus* in the family *Luteoviridae* includes at least 31 virus species that are exclusively transmitted by aphids in a circulative and persistent manner [103]. However, two exceptions of this genus were recently reported to be transmitted by the whitefly. A new recombinant of pepper vein yellows virus (PeVYV) reported is Israel was shown to be transmitted by the whitefly MEAM1, and not by aphids, and thus named pepper whitefly-born vein yellows virus (PeWBVYV). An additional recombinant of cucurbit aphid-borne yellows virus (CABYV) was reported to be transmitted by the whitefly MEAM1 in Brazil and then named cucurbit whitefly-borne yellows virus [10,104].

Thus, this paper reviews the main whitefly-transmitted viruses (Table 1), which were organized by families and/or groups of plants of agronomic importance as a crop (i.e., solanaceous, legumes, cucurbitaceous) or non-cultivated plants (i.e., weeds and wild hosts) acting as a reservoir of viruses.

### 3.1. Whitefly-Borne Viruses Infecting Solanaceous

The first report of whitefly-borne viruses in solanaceous species occurred in tomatoes from Brazil in the 1950s [105]. By 1975, other tomato-infecting begomoviruses associated with *B. tabaci* were reported in the country, including tomato golden mosaic virus (TGMV), the first tomato begomovirus characterized in the Americas [106]. However, infections by these begomoviruses never caused economic losses, probably because of the low efficiency of the existing species *B. tabaci* NW in colonizing solanaceous plants [107].

The emergence of begomoviruses outbreaks in solanaceous species, mainly in tomato, across South America was directly associated with the introduction and rapid spread of *B. tabaci* MEAM1 in the early 1990s [11]. This introduction could have also facilitated the transference of indigenous begomoviruses from non-cultivated hosts to tomato. Since then, a large number of new begomoviruses infecting tomatoes have been characterized [108,109,110,111,112,113,114,115,116,117,118,119,120]. This fact is reported mainly in Brazil, and evidence indicates that *B. tabaci* MEAM1, which unlike NW, colonizes easily solanaceous species and transmits indigenous begomoviruses previously restricted to wild hosts to tomato, contributing significantly to the rapid emergence and establishment of these viruses in the country.

**Figure 2:**
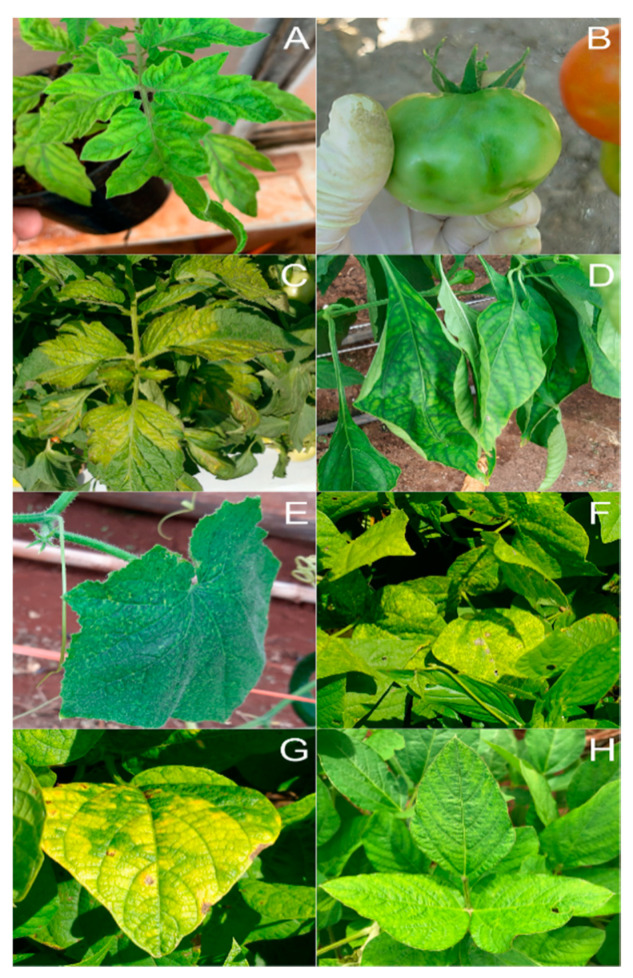
Symptoms associated with whitefly-transmitted viruses. (**A**) Symptoms of leaf rugose and internerval chlorosis caused in tomato leaf by tomato severe rugose virus. (**B**) Fruit deformation in tomatoes infected with tomato yellow vein streak virus. (**C**,**D**) Yellowing symptoms are caused by tomato chlorosis virus in tomato and bell pepper leaves, respectively. (**E**) Mottling symptoms in cucumber infected with tomato chlorosis virus. (**F**) Symptoms of mosaic in beans infected with bean golden mosaic virus. (**G**,**H**) Symptoms of cowpea mild mottle virus in common beans and Soybean, respectively.

Currently, 18 species of begomovirus have already been described naturally infecting tomato in Brazil. Despite the diversity of viruses detected, few species are widely distributed throughout the country, and most of them are restricted to certain regions [109,113]. Tomato severe rugose virus (ToSRV) is the most prevalent begomovirus in Brazil (Figure 2A), mainly in the Midwest and southeastern states [113,118,121,122], whereas tomato chlorotic mottle virus (ToCMoV), tomato common mosaic virus (ToCmMV), and tomato yellow vein streak virus (ToYVSV) have also been found frequently in the southeastern states [116,117,123].

In general, most tomato-infecting begomoviruses in the New World (NW) are bipartite and induce similar symptoms including mosaic, mottle, yellowing and vein-clearing chlorotic lesions, rugosity, leaf curling, and growth reduction [124]. Curiously, an NW monopartite begomovirus reported in Peru in 2011 [125] induced different symptoms, including curling and deformation of leaves, stunting, and distorted growth [110,125]. This virus, denominated tomato leaf deformation virus (ToLDeV) [125], has also been found in Ecuador [110], North of Chile [126], and with incidences up to 100% in northern and central Peru, causing significant losses [110,125,127]. Another NW monopartite begomovirus species denominated tomato mottle leaf curl virus (ToMoLCV), commonly found in the northeastern states and the northern region Minas Gerais in Brazil, induces similar symptoms. [11,25,128]. These reports indicate that the *B. tabaci* super vector may drive the emergence of monopartite begomoviruses native of the Americas [11].

All tomato-infecting begomoviruses reported until now in South America are native, evolving from local viruses, except for tomato yellow leaf curl virus (TYLCV), an invasive Old-World begomovirus associated with the tomato yellow leaf curl disease (TYLCD), the most important and devastating tomato disease in the world. In South America, TYLCV has only been reported in Venezuela, exhibiting typical symptoms such as reduction, yellowing, and upward curling of leaves [111,129,130] in the most important tomato-growing areas of this country [111]. However, although no other reports of TYLCV in other South American countries published yet, the occurrence of MED in neighboring countries, such as Brazil, points to the need for TYLCV monitoring in South American countries.

In addition to TYLCV, potato yellow mosaic virus (PYMV) and merremia mosaic virus (MeMV) are the more prevalent begomoviruses in Venezuelan tomato fields [111,131]. Other begomoviruses have also been reported in tomatoes in this country (Table 1) but occurring in lower frequency. In previous studies to assess the occurrence of tomato viruses in different states in the country, PYMV (previously described as tomato yellow mosaic virus, ToYMV) was the most prevalent begomovirus [111,131]. This virus has been associated with tomato plants in Venezuela since 1963 [132], and it has also been the most important begomovirus in tomatoes in Colombia [51,112] and several Caribbean basin countries [133,134]. The spread of this virus is possibly associated with a high incidence of *B. tabaci* MEAM1 in Venezuela [135] and Colombia [136,137].

In the last years, an increase in the number of emergent begomovirus reports has occurred in tomato-producing regions in Peru, Uruguay, Argentina, and Chile. In Peru, in addition to ToLDeV, another novel begomovirus named pepper leafroll virus (PepLRV) was reported in tomato, but in low incidence rates and showing latent infection [138]. Tomato rugose yellow leaf curl virus (ToRYLCV) and ToYVSV are the begomoviruses found infecting tomato crops in Uruguay [136,139]. Although the first report of ToRYLCV was in 2014, the disease symptoms have been observed in the northern of the country since 2005. Both viruses are also found in Brazil [136].

Brazil, Argentina, and Chile are the largest fresh tomato producers in South America [140]. Typical symptoms of begomovirus infection are often found in tomato fields throughout these countries; however, in Argentina and Chile, many of these begomovirus species are in the process of identification and molecular characterization.

Surveys conducted between 2009–2015 in north Chile’s main tomato-producing regions showed that ToYVSV (Figure 2B) is the predominant begomovirus, followed by ToLDeV [126,141]. There is neither report of detecting these viruses nor its whitefly vectors in other regions in the country.

In Argentina, two new species, denominated tomato dwarf leaf virus (ToDLV) and tomato mottle wrinkle virus (ToMoWrV), were characterized in tomato in 2012 and 2015, respectively [142,143]. Two other new species were recently identified [144,145], but ICTV has not yet recognized them.

In potato, three species of begomovirus have already been detected in South America: ToYVSV, ToSRV (Brazil), and PYMV (Venezuela). ToYVSV causes yellow mosaic and leaf distortion in potato [146]. ToSRV infecting potato plants in Brazil was observed in 2008 [147]; however, potential damage to the crop and economic losses have not been reported [25,148]. PYMV was first described in Venezuela, 1963, in potato plants that grew close to infected tomato crops. Symptoms in potato include bright yellow mosaic, leaf distortion, and stunting [149,150].

Begomoviruses do not represent a threat to Brazil’s potato production, but they may act as an inoculum source for tomatoes and other solanaceous species [148]. Potato is vegetatively propagated, and the virus may be translocated to tubers, producing infected seed tubers with reduced quality [25].

The crinivirus ToCV is one of the most important viral diseases of tomato (Figure 2C), potato, and sweet pepper plants (Figure 2D) worldwide. In South America, ToCV has only been described in Uruguay [151] and Brazil [152]. First, the ToCV was identified in tomato plants [152], followed by the identification in sweet pepper [153], potato [154], eggplant, scarlet eggplant [155], and recently infecting cucumbers [156] in Brazil (Figure 2E).

Although ToCV has a wide range of plant hosts, symptoms observed by virus infection on several infected plants mainly include older/lower leaves with an interveinal yellowing and thickening of leaves that may gradually progress to younger leaves. However, the symptoms are also similar to physiological or nutritional deficiency, making worrisome the correct disease diagnosis [157,158]. Additionally, it is common to find asymptomatic infections in sweet pepper, but these still can have yield reduction and smaller sizes when compared to healthy ones [157].

Nowadays, ToCV can be found mainly associated with tomato plants in practically all South, Southeast, and Northwest regions of Brazil. Nevertheless, incidences reaching >90% of ToCV in tomato crops were reported in the Midwest regions of Brazil, where successive tomato fields are implemented, maintaining high whitefly infestations [159]. Other high incidences of ToCV were observed in greenhouses with tomato and sweet pepper in the state of São Paulo, with incidences of MED reaching 100%.

Another important crinivirus in South America is the potato yellow vein virus (PYVV), the causal agent of potato yellow vein disease in Colombia, Venezuela, Peru, and Ecuador [160,161]. PYVV is exclusively transmitted by *T. vaporariorum* [161]. Moreover, some studies have shown that the movement of infected seed tubers might be the main mechanism of dispersion and could be a key driver for PYVV infection among potato crops [162]. The experimental host range of potato yellow vein virus is quite restricted, including potato, tomato, and some species of weeds [44,161].

Regarding poleroviruses, PeVYV was recently reported infecting bonnet pepper plants in Brazil [104]; however, no associated vector was identified yet. The recent report of a whitefly-transmitted recombinant of PeVYV, reported in Israel [9], highlights the possibility of having whiteflies associated with this new polerovirus in Brazil, encouraging the necessity of further studies to characterize damage and associated vectors.

### 3.2. Whitefly-Borne Viruses Infecting Legume Crops

The first viral disease reported to be of epidemiological and economic importance in South America was caused by the begomovirus bean golden mosaic virus (BGMV), infecting common bean in São Paulo, Brazil [163]. This disease is characterized by a remarkable golden yellow mosaic and chlorosis on leaves, with plants frequently showing dwarfism, especially when infected in early stages, reducing yields substantially (Figure 2F) [164]. Right after its report, in one decade, the bean golden mosaic, as it is known, was rapidly disseminated to other states of Brazil [165] along with the expansion of soybean cultivation in the 1970s in Brazil and Argentina. In Argentina, besides BGMV, bean dwarf mosaic virus (BDMV) also became a limiting factor for common bean production. Currently, BGMV is the most economically important bean-infecting virus in Brazil and Argentina, with losses up to 100% during the dry season [124,166].

Tomato begomoviruses can infect not only solanaceous plant species, but also legumes such as common and lima bean and soybean. Recently, it was shown under experimental conditions that asymptomatic common bean and soybeans infected with ToSRV can be potential amplifier hosts for these viruses and vectors to a nearby tomato crop, contributing even further to the begomovirus epidemic in Brazil [167]. The ToSRV was also reported infecting common bean and soybean in central Brazil, but the incidence in both crops was lower than 3.5%, and all infected plants were asymptomatic. However, a soybean area with >10% incidence of asymptomatic ToSRV-infected soybean plants was found in the southeast of Brazil (Sao Paulo State) in 2018 [167]. However, the impacts on bean and soybean production caused by ToSRV are unknown.

In Argentina, BGMV and the soybean blistering mosaic virus (SbBMV) are begomoviruses found infecting bean and soybean plants [166,168]. The tomato yellow spot virus (TYSV-AR), previously known as sida mottle virus (SiMoV), isolated from Argentina, can also infect beans and soybean plants [169]. Begomoviruses are more common on beans compared to soybean in Argentina, being mainly found in soybean in the Northeast region [169]. Among these begomoviruses in Argentina, the BGMV is the most frequently found infecting bean plants [168]. Recently, three other begomoviruses were described naturally infecting beans: the tomato mottle winkle virus (ToMoWV), sida golden mosaic Brazil virus (SiGMBRV), and ToYVSV. The last two are well known in South America, whereas ToMoWV is a new begomovirus that seems to be the result of a recombination between SbBMV and ToYVSV [166]. This new begomovirus has become the most frequent virus in beans production areas in Argentina, with incidences higher than 60% [166].

In Ecuador, the begomovirus cabbage leaf curl virus (CabLCV) was reported infecting beans (common bean and cowpea), but not in soybean [170]. A novel strain of pepper leafroll virus (PepLRV) was also reported infecting common bean and soybean in Ecuador [171].

Other begomoviruses were reported infecting bean in South America countries, such as PepLRV infecting beans in Peru [138], the bean leaf crumple virus infecting common bean in Colombia [172], as well as the bean yellow chlorosis virus (BYCV) and bean white chlorosis mosaic virus (BWCMV) infecting common beans in Venezuela [173]. However, no additional information other than the reports is available to date.

The carlavirus CPMMV was first reported in the Americas in 1983, infecting common bean fields (Figure 2G) in Brazil [174] and later soybeans in the early 2000s (Figure 2H), becoming a common disease in this crop since them. CPMMV symptoms observed in soybean genotypes used in the 2000s included mosaic, leaf crinkle, leaf blistering, dwarfism, bud blight, and vein clearing that progress to necrosis with time, giving the disease the common name of “stem necrosis of soybean” [175]. The more recent soybean genotypes used in Brazil when infected with CPMMV can present mottle or be symptomless [176,177]. Recent studies revealed that some soybean cultivar could be affected in productivity even when the CPMMV infection are symptomless [178]. After three decades since its first report in common bean in Brazil, CPMMV has emerged again in 2013 in common bean fields. This re-emergence of CPMMV occurred especially in the BGMV-resistant GM common bean lines once the golden mosaic symptom disappeared in the genotypes [179].

The continuing presence of CPMMV in soybean and bean in Brazil demonstrate the real challenge to manage this disease, especially in areas where the crop rotation soybean–bean are common as in Goiás, Paraná, and São Paulo State, for example, where this system facilitates the maintenance and spread of the virus. CPMMV in weeds can increase virus incidence in different areas besides soybean and common bean fields, bringing the necessity of identifying new possible natural hosts and reservoirs of this virus. Along with the effective transmission of CPMMV by whiteflies [97,98,99,180], the virus dispersion through South America, mainly in Brazil, has been likely facilitated by the occurrence of asymptomatic infections and the international trade of infected seed, which is quite feasible [178,181].

Besides Brazil, Venezuela is the only country in South America where CPMMV was reported infecting a legume crop. Yardlong (*Vigna unguiculata* subsp. *sesquipedalis*) is a subspecies of the cowpea, and CPMMV can be considered a real threat for this crop in Venezuela [182].

### 3.3. Whitefly-Borne Viruses in Cucurbitaceous

“Melon yellowing” is the primary viral disease of melon plants in Brazil [10,25]. This disease is often found in mixed infections with at least three viruses infecting melon plants, such as CABYV, transmitted by aphids, and CWBWV, which, like MYAV, is transmitted by the whitefly *B. tabaci* MEAM1 species [10,183]. Symptoms caused by virus infection on plants mainly develop on older leaves with yellowing and mottling, as well as the reduction of solid soluble content (°Brix) in the fruits [10]. The MYaV and CWBWV were reported experimentally infecting some zucchini and West Indian gherkin species, while cucumber and watermelon seem resistant to these viruses [10,25]. These viruses (MYaV and CWBYV) were reported only in the Northeast region of Brazil, where high incidences of the disease are frequent in almost all field-grown melon and usually coincide with the high whitefly infestation [10,25].

In Venezuela, the melon chlorotic mosaic virus (MeCMV) is the main begomovirus that causes a significant disease that affects melon and watermelon crops [5,6]. This disease is characterized by a severe and mild symptom on young melon and watermelon leaves, respectively [184]. The MeCMV was also reported naturally infecting zucchini, cucumber, and some wild cucurbits. To date, its incidence can reach 93% on melon and 83% on watermelon crops [184].

### 3.4. Whitefly-Borne Viruses in Non-Cultivated Plants

Several weeds are known hosts of viruses from *Begomovirus*, *Crinivirus,* and *Carlavirus* genus. Wild plants are commonly found infected by begomoviruses, and spillover to crops has occurred. On the other hand, spill back from crops to wild plant hosts can also occur and result in new outbreaks of begomoviruses [185]. Weeds, such as *Datura stramonium*, *Euphorbia heterophylla*, *Sida* spp., *Nicandra physaloides*, *Malva* spp., and *Crotalaria* spp., are known hosts of ToSRV, the most widespread and important from the genus *Begomovirus* infecting tomato in Brazil [186,187,188]. Species as *N. physaloides* were shown to be efficient reservoirs of ToRSV and sources of inoculum for viral reinfection in tomatoes after a crop-free period situation [189]. Some begomoviruses such as sida micrantha mosaic virus (SiMMV), sida mottle virus (SiMoV), and sida common mosaic virus (SiCmMV) have also been reported infecting tomato plants [118]. Similarly, begomoviruses detected in tomato can also be found infecting wild plants [117,126,187,190]. A wide range of weeds can also be infected by ToCV, including common weeds in the field like *Solanum americanum*, *N. physaloides*, *Amaranthus viridis*, *Chenopodium album,* and many others [191,192]. The carlavirus CPMMV was also detected in several uncultivated species, including *Sida* sp., *Macroptilium* spp., *Senna* spp., *Desmodium glaborum*, *Rhynchosia minima*, *Mirabilis jalapa*, *Cleome affinis*, and *Blainvillea rhomboideia* [193].

## 4. Whitefly Management

Whiteflies are one of the hardest insects to manage in many crops around the globe, because they have different behaviors and preferences. These insects can coexist in several common cultivation systems, but in some cases, interspecific competitions in the same host may occur, being influenced by whitefly biological characteristics and environmental conditions. Studies conducted in China comparing the indigenous *B. tabaci* species Asia II 1 and the invasive MEAM1 indicated that, in general, MEAM1 has a wider host range that consequently results in a better capability in its invasion process [194]. Another point related to population interactions of *B.tabaci* cryptic species is how insecticides can influence their dynamics. In China, researchers noted a rapid replacement of *B. tabaci* MEAM1 by MED because of the intense use of insecticides, correlating it with the occurrence of TYLCV outbreaks [195].

Referring to the wide adaptability of this cryptic species in many different hosts, *B.tabaci* outbreaks are commonly noticed all over the globe, and since the early 19th century, this species has been spreading to new countries, such as India [196], Mexico [197], Israel [198], United States [199], and others. In the past decades in Brazil, sporadic *B. tabaci* outbreaks were reported, and these infestations were probably related to indigenous species of the New World group [55]. However, since the 1990s, the main concern is related to the cryptic species MEAM1, which possibly displaced the native ones [56], which can change now, after the introduction of MED [67].

Studies carried out in Brazil have shown that the adaptation of *B. tabaci* MED and MEAM1, in a competitive scenario free of insecticides spraying, varied according to the host. *B. tabaci* MED became the dominant species compared to MEAM1 in sweet pepper (*Capsicum annuum* L.) and beans (*Phaseolus vulgaris* L.), after 120 days; however, in cotton (*Gossypium hirsutum* L.) and soybean (*Glycine max* (L.) Merr.), there was coexistence between the two species. In contrast, in tomatoes (*Solanum lycopersicum* L.), *B. tabaci* MEAM1 was dominant [85]. Reports made in the region of Colombia’s Vale do Cauca, in 1997, showed that the species *T. vaporariorum* and *B. tabaci* native (NW) were predominant. After the invasion of *B. tabaci* MEAM1 in the region, it was observed that these native species were displaced by the invasive [200].

In-depth knowledge of the pest characteristics, such as behavior, biology, physiology, and ecology, is an important step for developing integrated pest management (IPM) strategies. IPM allows decision-making based on individual and integrated control approaches, balancing the cost/benefit and taking into account the benefits for farmers, the environment, and society [201]. The IPM can contribute to environmental conservation and natural enemy preservation or enhancement [202] and is often based on the pillars of cultural, biological, and chemical control tactics.

### 4.1. Whitefly Management: Cultural Control

Cultural control can be a great alternative in which the environment modification can positively or negatively impact pest infestation in the cultivated area. Studies have shown that the nitrogen content in a plant can increase *T. vaporariorum* egg production, permitting the insect to obtain the necessary nutrients for its development and oviposition in a shorter feeding period time [203]. Water stress and the type of irrigation used in the area can also influence whitefly dynamic [204]. Studies conducted with cantaloupe suggested that daily sprinkler irrigation resulted in lower whiteflies infestation [205] and that the combined use of less susceptible cultivars to the insect as well as anavoidance of plant water stress can result in a lower number of whiteflies immatures on cotton plants [206].

Another cultural practice that has been adopted, which has been extremely important for reducing the whitefly population and the incidence of viruses in certain areas, is implementing free periods for specific crops. For example, in Brazil, a law implemented since 2014 by the Ministry of Agriculture, Livestock, and Supply (MAPA) prohibits bean cultivation for one month in certain areas of Goiás, Federal District, and Minas Gerais to reduce transmission and spread of BGMV. This practice was adopted in Brazil as an extreme action to avoid whitefly outbreaks, especially in critical periods of the year in which large areas of bean crops become a “reservoir” for re-invasion of other crops such as tomatoes, for example [124]. This practice required a continuous inspection from government inspectors, making sure that farmers followed the rules.

Due to whiteflies being polyphagous, they can survive and complete their cycle in many different hosts. For example, weeds are important plants to keep the whitefly population and serve as an inoculum source of whitefly-transmitted viruses [189]. Then, it is crucial to eliminate weeds from the surrounding areas to have effective management. One interesting option for reducing the incidence of whiteflies are mulching techniques. Studies testing different types of mulches indicate a reduction in whiteflies and virus incidence [207,208]. In many regions, another key point that should be considered a strategy in the IPM is the proper destruction of remaining crops after harvesting to avoid the migration of whiteflies to other crops and reduce virus outbreaks [209].

Another important aspect is that many crops are grown simultaneously in different regions of the continent. It is essential to understand that these insects can migrate from a crop to another, being responsible for disease outbreaks, especially when they are close and in different development stages. Recent studies conducted in Brazil, in the State of São Paulo, noticed a 10% incidence of asymptomatic ToSRV infection in senescent soybean near to recently transplanted tomato crops in which their incidence of symptomatic ToSRV was 57–70%, supporting the hypothesis of soybean crops acting like an amplifier host for begomoviruses in tomato [167].

**Figure 3:**
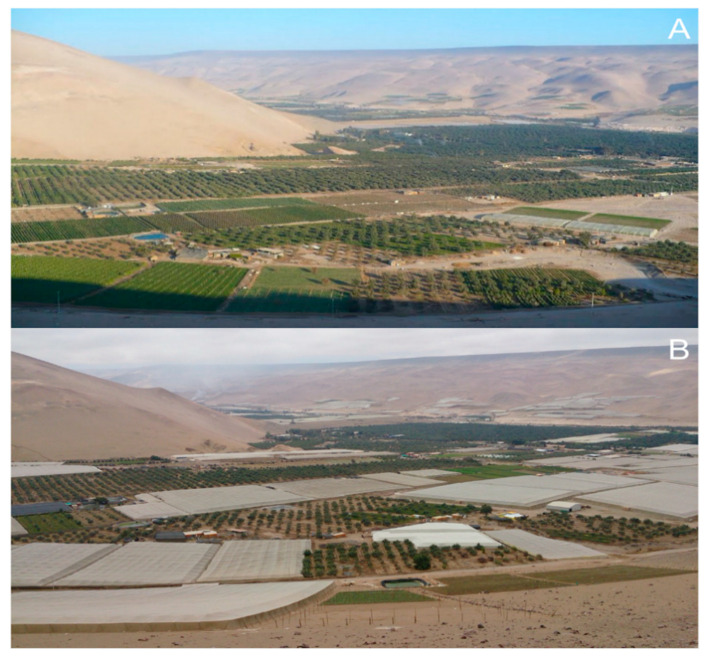
Evolution in adopting tomato cultivation under exclusion screens in Valle de Azapa, north of Chile, between years 2009 (**A**) and 2019 (**B**).

When we refer to protected crops, it is very convenient to use a proper barrier to prevent whiteflies infestations from outside areas. Therefore, insect screens have been adopted as a preventive exclusion method to stop vectors from entering the greenhouse. Although the right material acquisition can be expensive, especially for small farmers, it is very useful to prevent infestations. For *B. tabaci*, the maximum role size for a mesh suggested is 462 μm [210]. These screens are very reliable and environmentally safe, fit well into integrated pest management programs, and significantly reduce the need for insecticidal control. These materials are in widespread use in Israel, where, by the year 2000, practically all table tomatoes were grown under exclusion screens [211]. Similarly, in the north of Chile, fresh tomatoes’ production is being conducted mostly under exclusion screens, which is an economically viable pest management method (Figure 3) [74].

The use of repellent plants can also reduce the number of insecticide spraying, or even in organic crops. Studies conducted in Costa Rica have shown that intercropping with tomatoes and coriander plants (*Coriandrum sativum* L.) in open fields caused a reduction in whiteflies’ incidence and plants with viruses [212]. Other tests carried out in the Federal District region of Brazil, in tomato crops associated with coriander plants and sprinkler irrigation systems, have shown promising results in reducing the infestation of *B. tabaci* and incidence of damage caused by begomoviruses in periods of greater susceptibility of tomato culture [213]. More studies are still necessary to establish the best spacing of these repellent barriers to increase their efficacy.

In addition to all the factors mentioned above, it is relevant that, for the implantation of a crop, it is extremely important to choose a material that meets the biotic and abiotic characteristics found in the planting regions, such as the climate, soil characteristics, and water, as well as the record of pests and diseases that can occur in the area. Therefore, especially in regions where the incidence of diseases related to insects’ presence, such as *B. tabaci* and *T. vaporariorum*, the search for cultivars that have characteristics of tolerance or resistance to these pests and possible diseases is of great value. Several studies of different cultivable plants have been conducted to discover new compounds and resistance mechanisms that aim to reduce the damage caused by whiteflies. Among them, tomato plants have been explored, in which studies conducted with different genotypes concluded that the presence of high levels of allelochemicals such as acyl sugars (AS) and zingiberene (ZGB) associated with the presence of the *Mi* gene might be related to greater resistance to whitefly [214]. Besides the searching of resistant genotypes for whiteflies, studies are conducted to generate resistant cultivars to diseases, and the BGMV resistant bean is an example of it, in which Brazilian researchers developed a transgenic common bean resistant to this virus [215].

### 4.2. Whitefly Management: Biological Control

Another aspect of great importance in whiteflies management is the presence of other living organisms that cohabit in that place; among them is the beneficial entomofauna, which plays an important role in maintaining the ecological balance in the systems where it is present. The insects that make it up are responsible for the population control of other arthropods, through predation or parasitism, for the pollination of cultivated or spontaneous species, and for acting as macro-composers of organic material, actively participating in the cycling of nutrients [216,217].

The high diversity of species in the agroecosystems indicates its greater balance, with a direct relationship between the complexity of the environment and the abundance of species that compose the entomofauna. These insects work as bioindicators, and based on fauna studies, inferences about the degree of environmental disturbance can be reached. Research conducted in Brazil, Venezuela, Colombia, and Ecuador showed a vast entomofauna of parasitoids, including main parasitoids of the genera *Encarsia* and *Eretmocerus* [218]. Studies conducted on tomatoes in greenhouses from Colombia comparing the control of *T. vaporariorum* by the parasitoids *Encarsia formosa* and *Amitus fuscipennis* concluded that, when there is no air conditioning, the local parasitoids *A. fuscipennis* performed well in controlling this pest [219]. Parasitoids of the genus *Eretmocerus* were also found in Argentina parasitizing *T. vaporariorum*, and preliminary studies showed that the use of this parasitoid integrated with *Encarsia formosa* may be an option to help in the management of this pest [220]. Studies conducted in greenhouse peppers in Argentina concluded that the parasitoid *Eretmocerus mundus* performed well, parasitizing nymphs of *B. tabaci* in certain climatic conditions [221].

Predatory arthropods also play an important role in the balance of the agroecosystem. Among organisms that prey on *B. tabaci* and *T. vaporariorum*, there are insects of the family coccinellidae, some families of predatory stinkbugs and other insect families as well as predatory mites [222,223]. Studies carried out with the species *Tupiocoris cucurbitaceous*, a predatory bug found in greenhouses in Argentina preying on *T. vaporariorum*, concluded that this insect may be a potential biological control agent for this pest [224]. In addition to parasitoids, entomopathogenic fungi, including *Isaria fumosorosea*, *Lecanicillium* spp., *Beauveria bassiana,* and *Aschersonia* spp. can be used for whitefly management (Ascomycota: Hypocreales) [225,226,227]. Studies conducted in Brazil concluded that *I. fumosorosea* CG1228 showed good attributes for the development of microbiological insecticides for the control of this pest [228]. Other studies also conducted under greenhouses in Argentina by using different species of entomopathogenic fungi of *B. tabaci* and *T. vaporariorum*, including *Lecanicillium lecanii* (Zimmerm.), L. muscarium (Petch), *L. longisporum* (Petch), *Isaria fumosorosea* Wize, and *I. javanica* (Frieder. & Bally), showed that, on *T. vaporariorum*, there was a variation in the mortality rate from 26.6% to 76.7%, where the species *I. fumosorosea* CEP 206 was the most lethal [229]. In Brazil, there are several commercial products already available for whitefly control, as *Beauveria bassiana* strain ESALQ PL63, *Paecilomyces fumosoroseus* (http://agrofit.agricultura.gov.br/agrofit_cons/principal_agrofit_cons).

Therefore, it is possible to assume that biological control is an excellent tool for whitefly management, especially when used with other control tactics and the individual characteristics of each region in which it will be placed are observed. Then, this control strategy aligned with the chemical control can act assertively to keep the population below the limit of economic damage to crops. In Arizona, the implementation of an integrated control program of *B. tabaci* in cotton was responsible for the reduction of 70% of foliar insecticide spraying, resulting in an economy of $200 million of control costs along 14 years. The management system was based on research-defined sampling and thresholds, the observation and conservation of natural enemies by the use of selective insecticides when necessary, and also the use of ecological selectivity by knowing the interactions between pest, natural enemy, and environment [230].

### 4.3. Whitefly Management: Chemical Control

The decision to use chemical control as a tool to reduce the number of whiteflies is directly associated with sampling populations in most of the cases. There are different methods to sample whiteflies in crops, and sticky traps, on-plant counts, and beat trays are commonly used [231]. The sampling processes are very important to show if the pest infestation is reaching the economic threshold. The economic threshold for whiteflies varies according to crops and can be controversial, especially in crops infected by viruses. For example, cotton studies suggests an economic threshold of six adults of *B. tabaci* per leaf [232]. On the other hand, for crops that are compromised by several viruses, the control is made through the spraying of chemical insecticides to prevent infestations of the insect [233]. For potato, it is recommended that the monitoring of whiteflies should be made twice a week to avoid high infestations of whiteflies [234]. For crops such as cotton, beans, and soybeans, due to their big areas, this monitoring should be intensified if necessary with the objective of reducing whiteflies’ migration ‘’clouds’’ to other crops.

The main chemical groups of insecticides used in the control of *B. tabaci* are the groups of neonicotinoids (acetamiprid, clothianidin, imidacloprid, thiacloprid, thiamethoxam), growth regulators (buprofezin, pyriproxifen) and acetyl CoA carboxylase inhibitors (spiromesifen) [235], and the most recent group of diamides (cyantraniliprole), butenolides (flupyradifurone), and sulfoximines. Other common insecticide molecules used over the years are the pyrethroids, such as bifenthrin, cypermethrin, and others, and due to their intense use, many resistance cases were reported [236]. Some insecticides can be a good alternative for integration with biological control. Selectivity studies conducted with *Encarsia formosa* concluded that the molecule piriproxyfen was harmless to the pupae and adult stage of the parasitoid [237]. Other trials made with the molecules sulfoxaflor, flonicamid, metaflumizone, spiromesifen, and spirotetramat concluded that these insecticides were harmless to the *Amblyseius swirskii*, a generalist predatory mite of *B. tabaci*, and other insects [238]. These results show that some of these molecules can be an excellent alternative in the IPM perspective. Although chemical control is a very important and necessary tool for the management of whiteflies, its use without criteria and knowledge can cause problems as any other control technique. On the other hand, when we understand the aspects of each one, it is possible to construct a correct recommendation including, for example, the interval between chemical application and parasitoids inoculation on the area, avoiding negative impacts.

Over the years, with the great development of agriculture, the use of phytosanitary products has become essential for the cultivation of food, since they work in the management of agricultural and forestry crops, acting with great efficiency in biological targets [239,240]. Phytosanitary products are one of the most effective strategies, but can negatively affect the community of natural enemies [241]. The inappropriate use of these products can lead to the disappearance of beneficial individuals [242] and also, in the case of pests, increase the speed of the development of resistance to insecticidal molecules [243,244]. The overuse of insecticides in some regions was responsible for the selection of resistant individuals. A study conducted in Brazil with different populations of *B. tabaci* MEAM1 revealed that some populations had a high resistance factor (RR) when compared with susceptible individuals, for the active ingredients chloranthranilprole (601.14), imidacloprid (239.0), lambda-cyhalothrin (1521.36), and spiromesifen (46,578.39) having the highest value [245]. In addition to *B. tabaci* management problems, researchers observed failure in the control of *T. vaporariorum* after the use of the active ingredient metamidophos, and for *B. tabaci* they found resistance to the active ingredients methomil, metamidophos, and cypermethrin in regions of Colombia and Ecuador [246].

When referring to chemical control, a new scenario has been designed in South America, and this has started since the recent invasion of the cryptic species *B. tabaci* MED in the continent. In Brazil, it has been establishing itself in sweet pepper in high infestations in the State of São Paulo [92] and has the potential to adapt and cause damage to bean, soybean, and cotton crops [91]. In China, since the arrival in 2003 [247], MED became the dominant cryptic species in the region [195]. Comparing the susceptibility between MEAM1 and MED to some insecticides, MED showed lower susceptibility to the insecticidal molecules imidacloprid, nitenpyram, and thiametoxam [248]. Thus, MED can also spread throughout South America, especially in a scenario with a large number of insecticide sprays, due to the lower susceptibility to some insecticidal molecules used in the chemical control of this pest.

Therefore, exploratory tests were also carried out with whitefly populations from Brazil, and the objective was to establish susceptibility lines for the main commercial insecticides used to control whiteflies in the country, except for sulfoxaflor, which is a recently introduced molecule in South America, and whiteflies populations were not yet exposed to this insecticide. From the collection and identification of MED populations detected in 2017–2018 in the State of São Paulo in the municipalities of Holambra (HL) (22°37′59″ S 47°03′20″ W), Cerqueira César (CC) (23°00′10.1″ S 49°09′38.9″ W), and Victoriana (VT) (22°46′42.5″ S 48°24′19.3″ W), the curves were drawn according to the methodology of the leaflet [249]. The relative toxicity (RT) was calculated to compare each active ingredient between the populations. For the highest LC_50_, the RT value was considered to be 1.00, and for the lower LCs_50_, the RT was calculated by dividing the highest LC_50_ by the lowest LC_50_ (Table 2).

The variations of lethal concentrations (LCs) between populations may be related to different factors, for example, the intrinsic characteristics of each group of individuals given by the genetic variability of populations of these insects. This variability has already been observed in adults of *B.tabaci* MED collected in different regions in China, and when these insects were exposed to insecticidal molecules such as sulfoxaflor, cyantraniliprole, thiamethoxam, acetamiprid, and others, they showed different levels of susceptibility [250].

Another factor that may be linked to this variation in susceptibility among populations should be related to the management adopted in certain areas, in which intensive application of a certain group of insecticides may cause the selection of resistant individuals, as observed in Brazil, Colombia, and Ecuador [245,246]. Other studies conducted with MED and MEAM1 species noticed differences between those cryptic species related to gene expression of P450 involved in insecticide metabolism [251] and other enzymatic differences related to Glutathione S-transferases (GSTs) [252].

Additionally, the relationship of secondary endosymbionts with the lower susceptibility of *B. tabaci* has been demonstrated in studies, reporting that the combination and density of the set of endosymbionts *Rickettsia*, *Wolbachia,* and *Arsenophonus* can play a role in the susceptibility of the pest to insecticidal molecules such as thiamethoxam, acetamipride, and imidacloprid [253]. The *Rickettsia* endosymbiont, isolated at higher frequencies, is also correlated with lower susceptibility to the molecules of imidacloprid, acetamiprid, and thiamethoxam [24].

Thus, we can conclude that for the assertive management of whiteflies, it is necessary to understand several factors, using in a technical and integrated way the different strategies of control for these pests, searching for new ways to understand the complex relationships that these insects have in the environments in which they are present.

## 5. Future Perspectives

A lot of progress has been made in the last couple of decades about the study of whitefly dynamics in South America, and several factors that might be driving this distribution are being identified. However, there are still many questions to be addressed, and much research remains to be carried out. The recent finding of *B. tabaci* being a complex of morphologically indistinguishable species with genetic diversity and several biological differences opened up a completely new universe of research possibilities. Each of the different species of the *B. tabaci* may have a unique interaction with a determinate virus species or even a particular relationship with certain isolates of the same virus species. These particular interactions between whiteflies and viruses extend to the plant hosts and the different bacterial endosymbionts that the whiteflies harbor. All these biotic factors previously mentioned are also influenced by the many abiotic factors such as climate, production system, and pest management programs, among others. Therefore, each environment is different, making it essential for each country or region to have a local research program to unmask the characteristics of the whitefly populations belonging to that environment.

Whitefly taxonomy is constantly changing, and the amount of mtCOI data being deposited on databases is rapidly increasing. Although this relatively simple method brings a wide and representative situation of the dynamics of whitefly worldwide, some problems related to this approach have been identified, such as erroneous sequences and difficulties to differentiate nuclear mitochondrial DNA segments (NUMTs) from genes in mitochondrial DNA (mtDNA) [59,254].

The recent findings in *B. tabaci* taxonomy also revealed that MED species is divided into four different groups (Q1, Q2, Q3, and African silver-leafing–ASL) based on genetic variability and that the ASL group is isolated reproductively from Q1 and Q2 [254]. The different MED groups also present differences in host plant range, where the Q1 is the most polyphagous among them, which might have implications in management [255]. This could be one of the reasons to explain why MED seems to be a major concern in some countries or areas compared to others. There is still a lack of information about the role, the predominance, and the importance of each of the MED groups in South America. What is known is that Brazil reported the presence of two MED groups, Q1 and Q2 [35].

Another field of whitefly research that has been advancing in the last years is genomics. So far, the nuclear genomes of two *B. tabaci* species, MEAM1 [256] and MED [257], have been sequenced. Additionally, at least 24 whitefly mitochondrial genomes are available from 11 different species including the *B. tabaci* complex, *B. emillae,* and *B. afer* [258,259]. Among them, only six mitogenomes were sequenced from South America specimens including MEAM1, MED, and NW species. To date, there is no whitefly nuclear genome from a South American specimen available. Full genomes are valuable references and are essential to understand fundamental biological novelties and to aid the development of new approaches for whitefly management. Additionally, with the advent of high-throughput sequencing (HTS), the amount of research data being published has been increasing at a rate never seen before. In contrast, the biological data still takes longer to be generated, and many studies are missing this point. It is essential that applied studies with a focus on the biological characteristics keep being performed synchronized with studies based on molecular traits.

Regarding the abiotic components, the temperature is the key environmental factor among the several variables that affect whitefly reproduction, development, and dynamics [260]. The potential climate change scenario expected for the next decades may completely change the distribution of whiteflies species in South America with an expansion of the pest southwards; this occurrence is currently restricted mainly by the low temperatures. Therefore, whiteflies could soon reach pest status in some zones of temperate climate from Brazil, Argentina, Paraguay, and Chile, where they currently are not a major concern. Many studies have been performed using species distribution models to predict whitefly distribution according to climate at a global level [261], in Europe [262] and, more recently, in Central and South America [263]. These studies are very relevant and can assist in the development of control strategies when there is a high suitability for the occurrence of *B. tabaci*.

Although much data is being generated in research, only a few are actually being shared with growers or somehow being applied at production farms. With the advance of technology, new tools and softwares are aiding to overcome this challenge and are allowing to shorten the distance between the academic world and the growers. The management of whitefly is highly challenging, and it is essential to combine different academic disciplines of research to achieve the best product in the field.

## 6. Conclusions

This review showed that whiteflies are important as pests and virus vectors, being present in almost all South American countries, with some factors, such as temperature, endosymbiont diversity, host plants, associated viruses, and management affecting their behavior and development. Currently, *T. vaporariorum* and *B. tabaci* MEAM1 still predominate in South America, with the first more commonly occurring in highlands and subtropical or temperate regions, whereas the second being more important in tropical areas. Despite being recently introduced and not yet present in all South American countries, MED has a great potential to spread and change the whitefly hosts and virus scenario since it has differences in preference for hosts, virus-vector interactions, and insecticide active ingredients susceptibility.

Whitefly transmitted viruses are important mainly in solanaceous, legume, and cucurbit crops. The list of whitefly-transmitted viruses is growing over time, and the whitefly can contribute to it by inoculating different viruses in the same plant, increasing the possibility of recombination and rearrangements and the appearance of new strains or species. In contrast, the virus infection can favor whitefly development by reducing plant defenses or make the host more attractive by altering its physiological characteristics.

Whitefly management is challenging and distinct from one area to another because it depends on the presence of particular factors such as the endosymbiont diversity, host plants, presence of viruses, and control measurements history. Thus, for an assertive management of whiteflies, several studies in the affected areas’ characterization must be carried out, and from those, control strategies must be planned. Management must be composed by integrating different practices (cultural, biological, and chemical control) in a regional perspective, searching for new ways to understand the complex relationships that these insects have in the environments in which they are present.

## Figures and Tables

**Table 1:** Main whitefly-transmitted viruses present in South America.

Species	Genome	Vector	Country	Main Natural Hosts
**Genus *Begomovirus***				
bean golden mosaic virus (BGMV)	ssDNA bipartite	*B. tabaci*	Brazil ^1^, Argentina ^2^	Bean ^1,2^, soybean ^1,2^, *Macroptilium* spp. ^1^
bean dwarf mosaic virus (BDMV)	ssDNA bipartite	*B. tabaci*	Argentina	bean
bean leaf crumple virus (BYCV)	ssDNA bipartite	*B. tabaci*	Colombia	common bean
bean white chlorosis mosaic virus (BWCMV)	ssDNA bipartite	*B. tabaci*	Venezuela	common bean
bean yellow chlorosis virus (BYCV)	ssDNA bipartite	*B. tabaci*	Venezuela	common bean
cabbage leaf curl virus (CabLCV)	ssDNA bipartite	*B. tabaci*	Ecuador	bean
melon chlorotic mosaic virus (MeCMV)	ssDNA bipartite	*B.tabaci*	Venezuela	melon, watermelon, zucchini, cucumber, cucurbits
merremia mosaic virus (MerMV)	ssDNA bipartite	*B. tabaci*	Venezuela	tomato
pepper leafroll virus (PepLRV)	ssDNA bipartite	*B. tabaci*	Peru^1^, Ecuador ^2^	common ^1,2^, bean ^1^, soybean ^1,2^ pepper ^1^, tomato ^1^,
potato yellow mosaic virus (PYMV)	ssDNA bipartite	*B. tabaci*	Venezuela ^1^, Colombia ^2^	tomato ^1,2^, potato ^1^
tomato bright yellow mosaic virus (ToBYMV)	ssDNA bipartite	*B. tabaci*	Brazil	tomato
tomato bright yellow mottle virus (ToBYMoV)	ssDNA bipartite	*B. tabaci*	Brazil	tomato
tomato chlorotic leaf curl virus (ToCLCV)	ssDNA bipartite	*B. tabaci*	Brazil, Venezuela	tomato
tomato chlorotic leaf distortion virus (ToClLDV)	ssDNA bipartite	*B. tabaci*	Venezuela	tomato
tomato chlorotic mottle virus (ToCMoV)	ssDNA bipartite	*B. tabaci*	Brazil	tomato
tomato common mosaic virus (ToCmMV)	ssDNA bipartite	*B. tabaci*	Brazil	tomato
tomato dwarf leaf virus (ToDfLV)	ssDNA bipartite	*B. tabaci*	Argentina	tomato
tomato golden leaf distortion virus (ToGLDV)	ssDNA bipartite	*B. tabaci*	Brazil	tomato
tomato golden leaf spot virus (ToGLSV)	ssDNA bipartite	*B. tabaci*	Uruguay	tomato
tomato golden mosaic virus (TGMV)	ssDNA bipartite	*B. tabaci*	Brazil	tomato
tomato golden vein virus (TGVV)	ssDNA bipartite	*B. tabaci*	Brazil	tomato
tomato interveinal chlorosis virus (ToICV)	ssDNA bipartite	*B. tabaci*	Brazil	tomato
tomato leaf curl purple vein virus (ToLCPVV)	ssDNA monopartite	*B. tabaci*	Brazil	tomato
tomato leaf deformation virus (ToLDeV)	ssDNA monopartite	*B.tabaci*	Peru, Ecuador, Chile	tomato
tomato leaf distortion virus (ToLDV)	ssDNA bipartite	*B. tabaci*	Brazil	tomato
tomato mild mosaic virus (ToMMV)	ssDNA bipartite	*B. tabaci*	Brazil	tomato
tomato mild yellow leaf curl Aragua virus (ToMYLCAV)	ssDNA bipartite	*B. tabaci*	Venezuela	tomato
tomato mottle leaf curl virus (ToMoLCV)	ssDNA monopartite	*B. tabaci*	Brazil	tomato
tomato mottle wrinkle virus (ToMoWV)	ssDNA bipartite	*B. tabaci*	Argentina	tomato, beans
tomato rugose mosaic virus (ToRMV)	ssDNA bipartite	*B. tabaci*	Brazil	tomato
tomato rugose yellow leaf curl virus (TRYLCV)	ssDNA bipartite	*B. tabaci*	Brazil, Uruguai	tomato
tomato severe rugose virus (ToSRV)	ssDNA bipartite	*B. tabaci*	Brazil	tomato, potato, common bean, pepper, eggplant, soybean
tomato twisted leaf virus (ToTLV)	ssDNA monopartite	*B. tabaci*	Venezuela	tomato
tomato wrinkled mosaic virus (ToWMV)	ssDNA bipartite	*B. tabaci*	Venezuela	tomato
tomato yellow leaf curl virus (TYLCV)	ssDNA monopartite	*B. tabaci*	Venezuela	tomato
tomato yellow margin leaf curl virus (ToYMLCV)	ssDNA bipartite	*B. tabaci*	Venezuela	tomato
tomato yellow spot virus (ToYSV)	ssDNA bipartite	*B. tabaci*	Brazil ^1^, Argentina ^2^	tomato ^1^, beans ^2^, soybean ^2^
tomato yellow vein streak virus (ToYVSV)	ssDNA bipartite	*B. tabaci*	Brazil ^1^, Uruguay ^2^, Chile ^3^, Argentina ^4^	tomato ^1,2,3^, potato ^1^, bean ^4^
sida golden mosaic Brazil virus (SiGMBRV)	ssDNA bipartite	*B. tabaci*	Argentina	bean
soybean blistering mosaic virus (SbBMV)	ssDNA bipartite	*B. tabaci*	Argentina	bean, soybean
**Genus *Crinivirus***				
tomato chlorosis virus (ToCV)	ssRNA bipartite	*B. tabaci*, *T. vaporariorum*, *T. abutiloneus*	Brazil, Uruguay	Tomato, sweet pepper, potato, eggplant, scarlet eggplant, cucumber
potato yellow vein virus (PYVV)	ssRNA bipartite	*T. vaporariorum*	Colombia, Venezuela, Peru, Ecuador	potato
**Genus *Carlavirus***				
cowpea mild mottle virus (CPMMV)	ssRNA monopartite	*B. tabaci*	Argentina ^1^, Brazil ^2^, Venezuela ^3^	Soybean ^1,2^, bean ^1,2,3^
melon yellowing-associated virus (MYaV)	ssRNA monopartite	*B. tabaci*	Brazil	melon
**Genus *Polerovirus***				
cucurbit whitefly-borne yellows virus (CWBYV)	ssRNA monopartite	*B. tabaci*	Brazil	melon

**Table 2:** LC_50_ and LC_90_ values in different populations of *Bemisia tabaci* MED collected in 2017–2018 for the insecticidal molecules sulfoxaflor, imidacloprid, acetamiprid, thiamethoxam, and cyantraniliprole.

Insecticide	Line	*N*	Slope (±SE)	LC_50_ (mg ai L^−1^) (95% FL)	LC_90_ (mg ai L^−1^) (95% FL)	X^²^ (df)	RT
Cyantraniliprole ***	CC	900	1.70 (0.15)	17.41 (13.28–21.66) a	98.05 (78.73–128.80)	9.17 (6)	1.00
	HL	800	1.74 (0.19)	10.08 (6.87–13.24) a	54.93 (43.52–74.35)	3.89 (4)	1.72
	VT	700	1.52 (0.32)	9.23 (0.85–17.09) a	63.78 (35.38–527.29)	7.07 (3)	1.88
Sulfoxaflor **	CC	800	1.74 (0.18)	106.15 (76.49–135.58) b *	573.53 (453.88–779.50)	5.25 (5)	1.74
	HL	1100	1.77 (0.10)	185.47 (162.44–211.12) a	977.91 (802.30–1239.00)	11.87 (8)	1.00
	VT	900	1.30 (0.11)	44.20 (30.91–58.61) c	421.22 (326.95–573.35)	5.73 (6)	4.19
Imidacloprid ***	CC	900	1.29 (0.19)	539.87 (283.16–826.78) a	5277.00 (3007.00–.00)	12.10 (6)	1.00
	HL	800	4.33 (0.97)	248.62 (134.86–318.48) a	490.94 (385.49–855.38)	15.76 (5)	2.17
	VT	600	2.19 (0.24)	180.68 (74.91–284.72) a	683.93 (433,49–1888,98)	6.12 (3)	2.98
Acetamiprid ***	CC	800	2.03 (0.25)	55.21 (36.00–78.23) a	236.17 (155.99–462.44)	13.21 (5)	1.00
	HL	800	1.84 (0.19)	15.30 (11.53–19.00) b	75.66 (60.46–101.76)	4.69 (4)	3.60
	VT	700	1.88 (0.18)	38.78 (30.86–47.29) a	185.63 (142.84–263.97)	2.68 (4)	1.42
Thiamethoxam ***	HL	800	3.80 (0.37)	377.79 (330.84–423.81) b	820.75 (714.79–983.88)	6.08 (5)	1.61
	VT	700	3.10 (0.52)	609.64 (520.33–690.86) a	1576.00 (1250.00–2443.00)	4.55 (4)	1.00

SE: standard error; LC_50_: lethal concentration of the active ingredient required to kill 50% of tested individuals. LC_90_: lethal concentration of the active ingredient required to kill; 90% of tested individuals. 95% FL: confidence interval; X^2^: a test of linearity of the dose-response curves. * For each insecticide, different letters mean that 95% confidence intervals did not overlap the confidence intervals (95%). ** Active ingredients used for less than 2 years in South America. *** Active ingredients used for more than 4 years in South America. RT: relative toxicity to a population calculated based on LC_50_. For the highest LC_50_ for each active ingredient the RT value = 1.00.
